# Tunable magnetic anisotropy of antiferromagnetic NiO in (Fe)/NiO/MgO/Cr/MgO(001) epitaxial multilayers

**DOI:** 10.1038/s41598-023-31930-z

**Published:** 2023-03-24

**Authors:** W. Janus, T. Ślęzak, M. Ślęzak, M. Szpytma, P. Dróżdż, H. Nayyef, A. Mandziak, D. Wilgocka-Ślęzak, M. Zając, M. Jugovac, T. O. Menteş, A. Locatelli, A. Kozioł-Rachwał

**Affiliations:** 1 Faculty of Physics and Applied Computer Science, AGH University of Science and Technology, Kraków, Poland; 2 Jerzy Haber Institute of Catalysis and Surface Chemistry Polish Academy of Sciences, Krakow, Poland; 3 SOLARIS National Synchrotron Radiation Centre, Jagiellonian University, Krakow, Poland; 4 Elettra-Sincrotrone Trieste S.C.P.A., Basovizza, Trieste, Italy

**Keywords:** Magnetic properties and materials, Surfaces, interfaces and thin films

## Abstract

We report on the magnetic properties of antiferromagnetic NiO(001) thin films in epitaxially grown NiO/MgO(*d*_*MgO*_)/Cr/MgO(001) system for different thicknesses of MgO, *d*_*MgO*_. Results of X-ray Magnetic Linear Dichroism show that together with an increase of *d*_*MgO*_, rotation of NiO spins from in-plane towards out-of-plane direction occurs. Furthermore, we investigated how the proximity of Fe modifies the magnetic state of NiO in Fe/NiO/MgO(*d*_*MgO*_)/Cr/MgO(001). We proved the existence of a multidomain state in NiO as a result of competition between the ferromagnet/antiferromagnet exchange coupling and strain exerted on the NiO by the MgO buffer layer.

## Introduction

Conventional spintronic devices utilize electron’s spin degree of freedom to store information in the ferromagnetic layer^1^. However, recent demonstrations of magnetotransport effects in antiferromagnets (AFMs) make them excellent candidates for usage as active elements in future spintronic devices^2^. AFMs offer several advantages over ferromagnetic counterparts, such as higher packing density due to the absence of stray magnetic fields, robustness against external magnetic field and a potentially higher operation speed as a result of terahertz spin dynamics^3–5^. Different methods were utilized to modify the spin structure in AFMs, i.e. magnetic, optical, electrical and strain manipulation^6^. Magnetic methods include the application of relatively large external magnetic fields to align magnetic moments of AFMs^7,8^ and indirect control of the AFM spin structure via interfacial exchange coupling with a ferromagnet (FM) in FM/AFM bilayer systems, where a relatively small magnetic field is needed^9–13^.

The FM/AFM bilayers containing antiferromagnetic NiO are considered as model systems to study ferromagnet-antiferromagnet coupling^14^. Previous studies do not provide a conclusive view on the relative orientation between magnetic moments of FM and AFM layers in FM/NiO structure. While for the ideal FM/AFM interface a perpendicular alignment of AFM and FM magnetic moments across the interface is expected^14^, experimental observations of collinear and non-colinear coupling have been reported for FM/NiO^15–19^. Recently, NiO has been proposed as an active element of spintronics devices. Demonstration of spin hall magnetoresistance^20–22^, current-induced switching^23–25^ and optical generation of ultrafast spin current in NiO/Pt bilayers^26^ opened a new path toward the realization of NiO-based spintronic devices.

NiO is a transition metal oxide with a rock-salt crystal structure. In the bulk, below its Néel temperature of T_N_ = 523 K, the magnetic moments of Ni^2+^ cations are ferromagnetically aligned within each (111) plane and antiferromagnetically aligned between neighboring (111) planes^27^. Previous works demonstrated that appropriate strain engineering and finite size effects could modify the magneto-crystalline anisotropy and influence the direction of the magnetic moments in ultrathin NiO layers^28^. It was found that compressive strain leads to an in-plane NiO spin alignment, while tensile strain preferentially stabilizes an out-of-plane orientation of AFM spins. Lattice distortion in NiO can be induced either by the growth of AFM on ferroelectric^29,30^ or lattice miss-matched substrates^31–34^. Representatives of the latter approach are epitaxial NiO/Ag(001) and NiO/MgO(001) systems. It was shown that compressive strain in a thin NiO layer grown on Ag(001) stabilizes in-plane AFM domains^31,35,36^ while out-of-plane direction of AFM spins is preferred in NiO grown on MgO(001) substrate (*a*_*Ag*_ = 4.086 Å < *a*_*NiO*_ = 4.176 Å < *a*_*MgO*_ = 4.21 Å)^32,37^. Additionally, some previous studies have reported that in thin NiO layers grown on MgO(001) substrate direction of AFM spins can substantially deviate from its bulk counterpart^25,38^.

In our recent work, we performed systematic studies on the magnetic properties of NiO in Fe/NiO/Cr/MgO multilayers^33^. We proved that NiO grown on wedge-shaped Cr buffer undergoes continuous strain-induced spin reorientation transition (SRT) from nearly out-of-plane to in-plane direction as the strains change from tensile to compressive(*a*_*Cr*_ = 4.07 Å < *a*_*NiO*_ = 4.176 Å < *a*_*MgO*_ = 4.21 Å). Furthermore, for ultrathin NiO layers, we demonstrated an orthogonal (spin-flop*)* coupling between Fe and NiO spins.

In the present approach, we combine strain and ferromagnetic proximity to tune the magnetic spin structure of NiO. While strain induced by the substrate allows tuning magnetic anisotropy of the AFM from its bottom interface, interaction with the ferromagnetic cover layer enables to influence the magnetic state of the AFM from its top interface. Our results show that the insertion of an MgO layer between NiO and Cr buffer in a NiO/MgO/Cr stack strongly influences the spin structure in the AFM layer. Together with an increase of MgO interlayer thickness, we noted rotation of the NiO spins towards the out-of-plane direction. Furthermore, we investigated the magnetic response of NiO spins to the FM capping layer. Comparison of measured and simulated angular dependencies of the X-ray Magnetic Linear Dichroism (XMLD) spectra indicates that a domain structure of NiO is driven by a change of Fe and MgO thickness in Fe/NiO/MgO/Cr.

## Experimental

**Figure 1:**
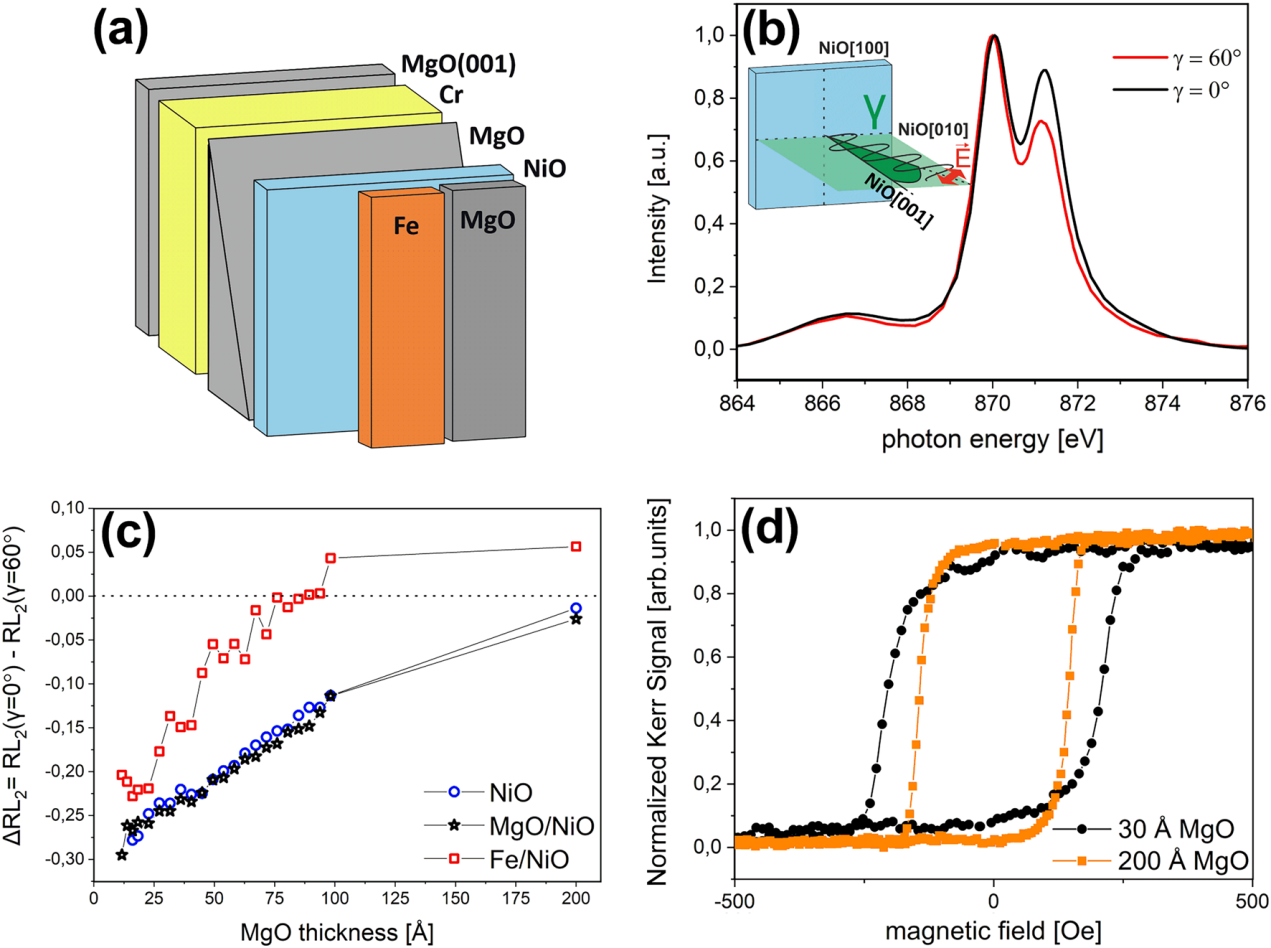
(**a**) Schematic sketch of the sample. (**b**) Exemplary Ni^2+^ L_2_-edge XAS spectra at γ = 0° (black line) and γ = 60° (red line) obtained at RT for NiO/MgO(18.3 Å)/Cr/MgO(001). The scheme in the inset shows the geometry of the XMLD experiment. (**c**) ∆RL_2_ ratio dependence on the MgO thickness in NiO/MgO(*d*_*MgO*_)/Cr (blue dots), MgO/NiO/MgO(*d*_*MgO*_)/Cr (black stars) and Fe/NiO/MgO(*d*_*MgO*_)/Cr (red squares). (**d**) LMOKE magnetic hysteresis loops acquired with external magnetic field along the Fe[100] direction at RT for Fe/NiO/MgO(30 Å)/Cr (black dots) and Fe/NiO/MgO(200 Å)/Cr (orange squares).

Samples were grown in an ultrahigh vacuum (UHV) chamber using molecular beam epitaxy on polished MgO(001) single crystals. The MgO substrates were annealed for 1 h at 753 K to obtain a clean surface before the deposition of thin films. A 50 Å MgO homoepitaxial layer was evaporated at 723 K using electron beam evaporation. Next, a Cr buffer layer was deposited at 473 K and annealed at 753 K to improve its surface quality. The Cr buffer thickness *d*_*Cr*_ was chosen to be 200 Å to ensure a fully relaxed surface^33^. Following Cr deposition, the MgO wedge-shaped layer with a thickness *d*_*MgO*_ in the range of (0–100) Å was grown at room temperature using a movable shutter and subsequently covered by a homogenous 20 Å-thick NiO layer. The NiO film was grown at room temperature (RT) by reactive deposition of Ni under the oxygen partial pressure of 1 × 10^−6^ mbar. At this stage, one-third part of the sample was capped with 20 Å of MgO and the other third part with 20 Å of Fe, which was additionally covered by a 30 Å-thick MgO protective layer. The MgO and Fe capping layers were evaporated at RT. As a result, three stripes with uncapped-, MgO-capped and Fe-capped NiO were formed along the MgO wedge (Fig. 1a).

The magnetic properties of NiO and Fe layers were characterized by means of X-ray Magnetic Linear Dichroism (XMLD) and X-ray Magnetic Circular Dichroism (XMCD), respectively. The X-ray absorption spectroscopy (XAS) measurements were performed at the PIRX beamline^39^ of the National Synchrotron Radiation Centre SOLARIS^40^. The XAS spectra were obtained in total electron yield (TEY) mode by measuring the sample current. The magnetic hysteresis loops were collected with longitudinal magneto-optic Kerr effect (LMOKE). A standard lock-in detection setup that consists of an s-polarized laser light source (λ = 635 nm) and a photo-elastic modulator with a modulation frequency of 50 kHz was used. The second harmonic signal (2f.) measured by the detector, which is proportional to the Kerr rotation, was taken as a measure of the magnetization. The photoemission electron microscope (PEEM) studies were performed at the DEMETER beamline of the National Synchrotron Radiation Centre SOLARIS and Nanospectroscopy beamline of the Elettra synchrotron (Trieste, Italy)^41^. In PEEM the X-rays are incident at an angle of 16° with respect to the sample surface. The XMLD-PEEM images were obtained by calculating the asymmetry of two images according to:

$$I_{asym} = \frac{{I(E_{1} ) - I(E_{2} )}}{{I(E_{1} ) + I(E_{2} )}},$$

where the energies E_1_ and E_2_ correspond to the two absorption peaks within the Ni L_2_ absorption edge (867.9 eV and 869.1 eV, respectively). The XMCD-PEEM images of Fe in the Fe/NiO/MgO/Cr structure were collected at the L_3_ absorption edge of Fe.

## Results and discussion

To investigate the magnetic properties of NiO in NiO/MgO(*d*_*MgO*_)/Cr, MgO/NiO/MgO(*d*_*MgO*_)/Cr and Fe/NiO/MgO(*d*_*MgO*_)/Cr multilayers, we performed systematic XMLD measurements as a function of the MgO thickness (*d*_*MgO*_). Figure 1b shows exemplary room temperature (RT) XAS spectra recorded at the Ni^2+^ L_2_ edge in the NiO/MgO(18 Å)/Cr for the incident angle γ of 0° (Fig. 1b, black) and 60° (Fig. 1b, red). The γ defines the angle between the sample surface normal and the propagation direction of the X-rays (see inset in Fig. 1b). After background subtraction, the spectra were normalized to the unity at the low energy peak. We noted a drastic change of the higher energy peak as a function of incident angle, i.e., the intensity of the peak at 871.2 eV increased for decreasing γ. The L_2_ ratio (RL_2_) defined as the intensity at the lower-energy peak divided by the intensity at the higher-energy peak is typically used to determine the spin orientation in NiO^42^. It is noteworthy that in the presence of a cubic crystal field, the XMLD effect is anisotropic. Therefore, the correct interpretation of XMLD results requires knowledge about the relative orientation of the X-ray polarization vector **E** and the spins with respect to the crystallographic axis^42,43^. X-ray linear dichroism (XLD) can originate not only from magnetic effects but also from the local crystal field effect. The crystal field effect contribution to the XLD signal in NiO is accompanied by an energy shift of the L_3_ edge between spectra acquired with grazing and normal incident X-ray angles^44^. In our studies, the XAS spectra acquired at the Ni^2+^ L_3_ edge do not reveal any energy shift as the incident beam γ angle changes from γ = 0° to γ = 60° (not shown). Consequently, we conclude that the XLD effect visible as a difference between two XAS spectra in Fig. 1b is of purely magnetic origin.

In our studies, the projection of the electric field vector on the NiO(001) sample plane, **E**_**ip,**_ was fixed to be parallel to the NiO[010] in-plane crystal axis (**E**_**ip**_ ║ NiO[010]). Such measurement geometry was utilized to probe the out-of-plane orientation of NiO(001) spins^32,37,45,46^. Figure 1c shows the dependence of the L_2_ ratio difference, defined as ΔRL_2_ = RL_2_(γ = 0°) – RL_2_(γ = 60°), on *d*_*MgO*_ for all three sample areas, i.e. (NiO/MgO(*d*_*MgO*_)/Cr (Fig. 1c, blue dots), MgO/NiO/MgO(*d*_*MgO*_)/Cr (Fig. 1c, black stars) and Fe/NiO/MgO(*d*_*MgO*_)/Cr (Fig. 1c, red squares). For both uncapped and capped NiO layers, we noted an increase of ΔRL_2_ together with an increase of *d*_*MgO*_. According to the previous studies^46^, an increase of ΔRL_2_ can be interpreted as an enhancement of the out-of-plane component of NiO spins. These results are supported by our LMOKE measurements performed for Fe/NiO/MgO(*d*_*MgO*_)/Cr. Figure 1d presents room temperature LMOKE hysteresis loops acquired for Fe/NiO/MgO(*d*_*MgO*_)/Cr for *d*_*MgO*_ = 30 Å (Fig. 1d, black dots) and *d*_*MgO*_ = 200 Å (Fig. 1d, orange squares). During the measurement, the external magnetic field was applied along the easy Fe[100] direction. We noted a significant enhancement of coercivity (H_c_) for the NiO grown on thinner MgO layer. While for *d*_*MgO*_ = 200 Å, a coercive field H_c_ = 143 Oe was registered, in the case of *d*_*MgO*_ = 30 Å, H_c_ = 206 Oe. For our samples, we did not note exchange bias, which suggests a perpendicular coupling between Fe and NiO. As previously shown, spin-flop coupling does not contribute to exchange bias but gives rise to a uniaxial anisotropy, which causes an enhancement of H_c_ in FM/AFM bilayers^47^. An enhanced Fe coercivity noted for thinner buffer confirms that for Fe/NiO/MgO(30 Å), for which we noted an in-plane direction of NiO spins, the uniaxial anisotropy is stronger than in Fe/NiO/MgO(200 Å) stack, for which an out-of-plane component of NiO spins is considerable. A similar tendency was presented in Fe/NiO/(Ag)/MgO for which, together with spin reorientation transition from in-plane to out-of-plane direction, a reduction of the coercivity was noted^32^. The results of LMOKE measurements confirm our interpretation of XMLD studies that the insertion of an MgO layer between NiO and Cr leads to the rotation of NiO spins toward the out-of-plane direction.

**Figure 2:**
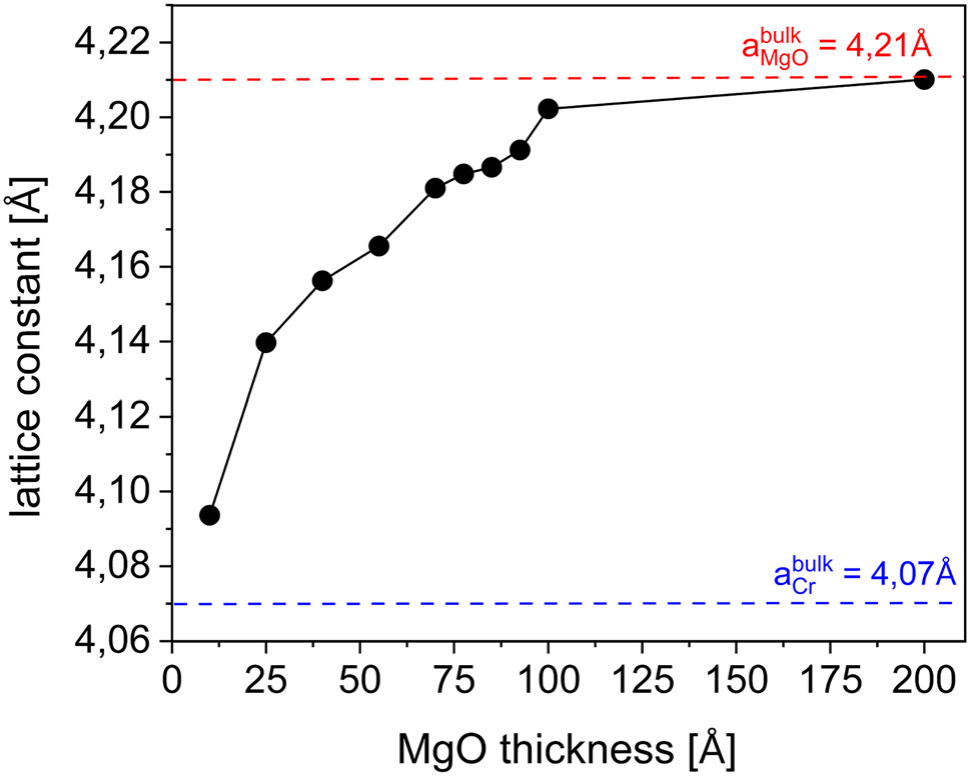
Lattice constant dependence on the MgO thickness in MgO(*d*_*MgO*_)/Cr(200 Å)/MgO(001), determined by analysing the LEED pattern collected across the MgO wedge.

To elucidate whether the spin reorientation transition (SRT) in NiO is related to the change of strain exerted by the MgO buffer layer, we analyzed the evolution of the MgO in-plane lattice constant as a function of its thickness. The MgO in-plane lattice spacing along the MgO[100] direction was determined from the low-energy electron diffraction (LEED) pattern collected across the MgO wedge for MgO(*d*_*MgO*_)/Cr(200 Å)/MgO(001) (Fig. 2). The data shows that MgO lattice constant* a*_*MgO*_ increases from *a*_*MgO[100*]_ = (4.09 ± 0.04) Å for *d*_*MgO*_ = 10 Å up to the bulk value of *a*_*MgO[100]*_ = (4.21 ± 0.04) Å for *d*_*MgO*_ = 200 Å. For thin MgO buffer layer, for which we noted pseudomorphic growth of MgO, a compression of NiO occurs (*a*_*MgO*_ = 4.09 Å < *a*_*NiO*_ = 4.18 Å) and AFM spins are aligned in plane, similar to NiO/Cr^33^ or NiO/Ag^32^. Together with an increase of lattice parameter of MgO, NiO experiences tensile stress (*a*_*MgO*_ = 4.21 Å > *a*_*NiO*_ = 4.18 Å), which prefers out-of-plane alignment of NiO spins^32,37^. Due to the charging of the surface, we were not able to perform systematic studies of the changes in the LEED pattern as a function of MgO thickness after the deposition of NiO. However, the analysis of the LEED pattern collected for MgO surface together with the analysis of XMLD results, brought us to the conclusion that strains determine the spin structure in NiO. The impact of strains on the magnetic properties of AFMs was theoretically studied by Finazzi and Altieri^28^. Theoretical calculations showed that tetragonal lattice distortions induce a change in the population of AFM domains via modulation of the dipolar energy, which dominates the magnetic anisotropy of NiO^28^.

Although we observed the same trends in ΔRL_2_(*d*_*MgO*_) dependencies for all three parts of the sample with different capping layers, a change of ΔRL_2_ as a function of MgO thickness is much more pronounced for the Fe/NiO/MgO(*d*_*MgO*_)/Cr region. To further examine how proximity to Fe influences the NiO spin structure in our system, we have performed a systematic XMLD study in Fe/NiO/Cr/MgO(001) and Fe/NiO/MgO(200 Å)/Cr/MgO(001).

**Figure 3:**
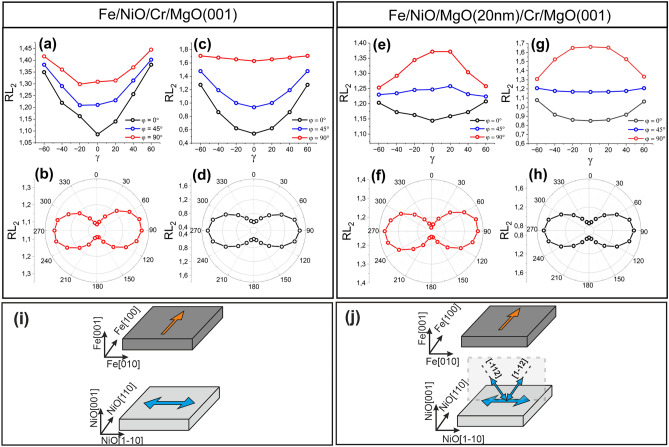
(**a**) and (**b**) Experimental Ni^2^^+^ RL_2_(γ) and RL_2_(φ) dependencies determined for Fe/NiO/Cr/MgO(001) system. (**c**) and (**d**) Calculated RL_2_(γ) and RL_2_(φ) dependencies for NiO spins aligned along NiO[1-10] axis. (**e**) and (**f**) Experimental Ni^2+^ RL_2_(γ) and RL_2_(φ) dependencies obtained for Fe/NiO/MgO(200 Å)/Cr/MgO(001) system. (**g**) and (**h**) Calculated RL_2_(γ) and RL_2_(φ) dependencies obtained for an equal population of selected in-plane and out-of-plane NiO domains (see the text for description). (**i**) and (**j**) A schematical illustration of NiO spin structure with directions of magnetic moments indicated by the arrows for Fe/NiO/Cr/MgO(001) and Fe/NiO/MgO(200 Å)/Cr/MgO(001), respectively.

Prior to XAS measurements, the sample was magnetized along the easy Fe[100] in-plane direction (NiO[110]) (see Figure S1, Supplemental Material for epitaxial relation between NiO(001) and Fe(001)). Fe L_2,3_ edge XMCD spectra acquired with two opposite helicities (Figure S2, Supplemental Material) confirmed that Fe magnetization **M**_**Fe**_ was aligned parallel to the applied magnetic field. In addition, XMLD studies were performed at the Ni L_2_ edge. Figure 3a depicts the RL_2_(γ) dependencies obtained for Fe/NiO/Cr for three different azimuthal angles φ, where φ is defined as the angle between the projection of X-ray polarization vector **E**_**ip**_ on NiO(001) plane and NiO[110] direction. For φ = 0° the **E**_**ip**_ is parallel to both the NiO[110] and the Fe magnetization, **M**_Fe_ (**E**_**ip**_║NiO[110]║**M**_Fe_), whereas for φ = 90° **E**_**ip**_║NiO[1–10]⊥**M**_Fe_. We noted the minimum of RL_2_ at γ = 0° for all the dependencies, which, based on some previous works^32,37^, indicates that the NiO spins lie within the surface plane.

In order to determine the azimuthal anisotropy of NiO spins in Fe/NiO/Cr/MgO(001), we performed XMLD measurements at normal incidence geometry (γ = 0°) as a function of the azimuthal angle φ. In such measurement geometry, a full in-plane sensitivity of XMLD to AFM spin orientation is available^32^. Figure 3b presents the RL_2_(φ) dependence for Fe/NiO/Cr with well-pronounced extrema for φ = 0° (**E**_**ip**_║NiO[110]║**M**_Fe_) and φ = 90° (**E**_**ip**_║NiO[1–10]⊥**M**_Fe_). According to the previous studies, if RL_2_ reaches an extremum when the polarization vector is parallel to the NiO[110] direction, the AFM spin alignment is determined from the maximum of RL_2_^32,48^. We noted the maximum of RL_2_ at φ = 90°(**E** ║NiO[1–10]⊥**M**_Fe_), which indicates that NiO spins are aligned parallel to the NiO[1–10] axis and perpendicular to Fe spins as expected for Fe/ultrathin NiO compensated interface (see Fig. 3i)^15,32,33^. In order to confirm our conclusions, we compared the experimental RL_2_ polar and azimuthal dependencies (Fig. 3a,b) with the theoretical ones (Fig. 3c,d).

To find theoretical spectra for a given relative orientation of the polarization vector **E** and the spins axis, we performed atomic multiplet calculations for NiO(001) using Crispy frontend^49^. The spectra were simulated for the atomic 2p^6^3d^8^ → 2p^5^3d^9^ transition in O_h_ crystal-field splitting of 10Dq = 1.4 eV^48^ and a total effective exchange field felt by the Ni^2+^ ion of 6 × 27 meV^44^. A detailed description of the simulations can be found in Supplemental Material. The calculated polar (γ) and azimuthal (φ) angles dependencies for NiO spins oriented along NiO[1–10] in-plane axis are presented in Fig. 3c,d, respectively. The shapes of calculated polar and azimuthal angles dependencies (RL_2_(γ) and RL_2_(φ), respectively) well reproduce the experimental data (Fig. 3a,b), which confirms the validity of our interpretation.

For Fe/NiO/MgO(200 Å)/Cr, we noted a well-defined uniaxial anisotropy with the maximum of RL_2_(φ) at φ = 90°(**E**_**ip**_║NiO[1–10]⊥**M**_Fe_) (Fig. 3f), similarly to the results obtained for Fe/NiO/Cr. However, the polar RL_2_ dependencies determined for Fe/NiO/MgO (Fig. 3e) differ significantly from the ones obtained for Fe/NiO/Cr (Fig. 3a). While for φ = 0° (**E**_**ip**_║NiO[110] ║ **M**_Fe_ ), the L_2_ ratio reaches the *minimum* at γ = 0°, for φ = 90° (**E**_**ip**_ ║NiO[1–10]⊥**M**_Fe_) the L_2_ ratio reaches the *maximum* at γ = 0°, whereas the L_2_ ratio remains almost constant for φ = 45° (**E**_**ip**_║NiO[100]). In order to explain the origin of RL_2_(γ) dependencies obtained for Fe/NiO/MgO, we compared experimental results with theoretical simulations. We simulated polar and azimuthal RL_2_ dependencies for all 12 possible bulk-like NiO domains (see Figure S3, Supplemental Material), obtaining a good agreement with the results presented in Ref.^50^. None of the theoretical dependencies obtained for bulk single-domain states of NiO reproduces our experimental results. This suggests that a multiple antiferromagnetic domain structure in NiO embedded between Fe and MgO should be considered. Therefore, before moving onto our results, we will introduce the possible magnetic domains in bulk NiO.

Below T_N,_ the antiferromagnetic order in bulk NiO results in a contraction of the cubic unit cell along [111] directions. Consequently, four twin domains (the so-called T domains) with contractions along different [111] directions are formed. Within each T domain, the spins can be aligned along one of the three [112] axes (S domains) which give rise to 12 possible different domain orientations in NiO^27,51^. As we consider NiO(001) plane, we can identify two distinct groups of domains that differ in the orientation of magnetic moments relative to the (001) plane. The first group with a large component of magnetic moment perpendicular to the surface ([11-2], [1–12], [-112], [112]) and a group of eight domains for which in-plane component of magnetic moment dominates ([1-21], [-121], [121], [10-1], [-211], [211], [21-1], [2-11])^51^.

The shape of experimental RL_2_(φ) dependence (Fig. 3f) and a possible orthogonal coupling between Fe and NiO indicate that magnetic domains in NiO exhibit a projection along an in-plane NiO[1–10] axis. In addition, a comparison of the experimental RL_2_(γ) dependencies (Fig. 3e) with simulated ones (Fig. 3g) excludes the existence of pure in-plane or out-of-plane spin orientation in NiO which indicates that both types of domains should coexist in the NiO layer. The shapes of RL_2_(φ) and RL_2_(γ) can be successfully reproduced in simulations where the coexistence of two bulk-like domains with a large out-of-plane component and projection to NiO[1–10] axis (i.e. [1-12] and [-112] magnetic domains) together with in-plane magnetic domain with spins oriented along [1-10] axis was considered. Figure 3j shows the schematical drawing of NiO domains which were considered in calculations of RL_2_(γ) and RL_2_(φ) (Fig. 3g,h).

RL_2_(γ) and RL_2_(φ) were calculated assuming an equal population of mentioned out-of-plane and in-plane NiO domains. We observed a change in the character of the RL_2_(γ) dependence for φ = 0° and φ = 90°, similar to the experimental results (compare Fig. 3e,g). This indicates that the appearance of the out-of-plane domains in NiO is related to the tensile strain exerted on the AFM layer by the MgO buffer, in contrast to the Fe/NiO/Cr/MgO for which the existence of [1-10] in-plane domains was sufficient to reproduce the experimental data. Although simulations well reproduce the shapes of the angular RL_2_ dependences, the amplitudes of theoretical L_2_ ratios differ from the experimental ones. This can be understood if we consider that the theoretical dependencies were calculated for the NiO bulk-like parameters. Moreover, the XMLD magnitude can be affected by experimental energy resolution^43^. Thus, we emphasize that a comparison between experimental and simulated L_2_ ratios should be treated qualitatively.

The exchange coupling between Fe and NiO spins at the interface, which was inferred from the XMLD and XMCD data, is directly confirmed by XMCD- and XMLD-PEEM measurements. In the PEEM setup, the X-rays were incident on the sample at 16° grazing angle from the surface. Therefore, the linear polarization can be either fully in the surface plane, giving sensitivity to the in-plane domain structure of NiO, or mostly out-of-plane, giving sensitivity to the out-of-plane NiO spin orientation.

**Figure 4:**
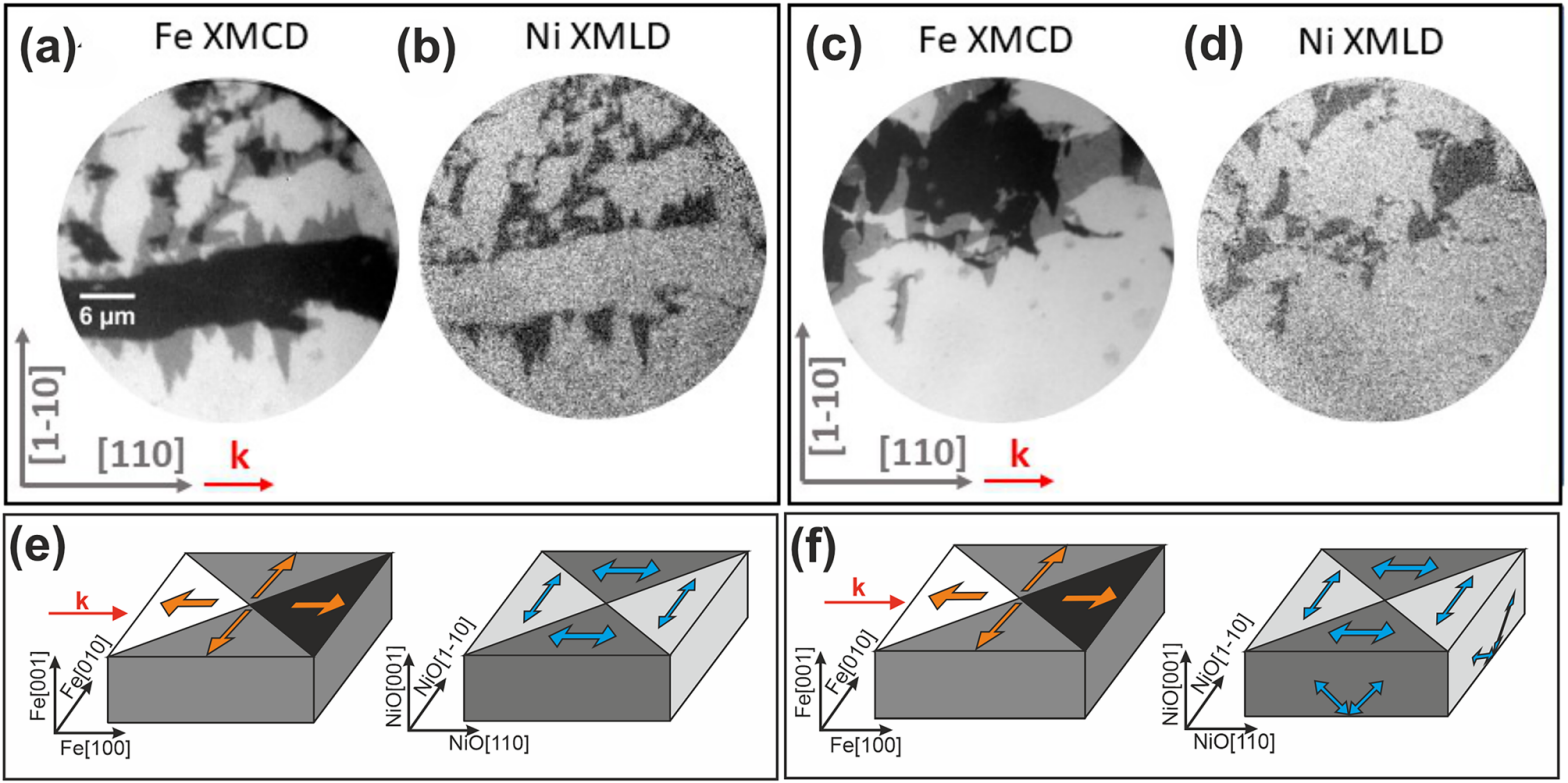
(**a**, **c**) The Fe L_3_ XMCD-PEEM images obtained for Fe/NiO/MgO(10 Å)/Cr and Fe/NiO/MgO(200 Å)/Cr, respectively. (**b**) and (**d**) corresponding Ni L_2_ XMLD-PEEM images acquired with vertical polarization. (**e**) and (**f**) a schematical illustration of spin structure with directions of magnetic moments indicated by the arrows for Fe/NiO/MgO(10 Å)/Cr and Fe/NiO/MgO(200 Å)/Cr, respectively.

Figure 4a,c represent the XMCD-PEEM images of the Fe magnetic domain patterns in Fe/NiO/MgO(10 Å)/Cr and Fe/NiO/MgO(200 Å)/Cr, respectively. The images were recorded after exposing the sample to an alternating in-plane magnetic field. The direction of the photon beam propagation **k** was set to be along to Fe[100] easy axis. Three contrasts can be distinguished in the XMCD-PEEM images. The dark and bright areas in Figs. 4a,c correspond to the antiparallel Fe domains magnetized along [100] direction, whereas the gray areas indicate the domains perpendicular to the **k** direction**,** which presents no magnetic signal. For both Fe/NiO/MgO(10 Å)/Cr and Fe/NiO/MgO(200 Å)/Cr the magnetic domain pattern of Fe is reflected in the NiO magnetic structure, as seen in the corresponding XMLD-PEEM images obtained with in-plane X-ray polarization (Figs. 4b,d). This proves that the FM/AFM coupling at the interface occurs in both systems. Magnetic contrast between the antiparallel Fe[100] domains is not visible in XMLD-PEEM images, as it is expected for antiferromagnetic NiO.

The NiO spin orientation within the domains presented in the XMLD-PEEM images can be determined by considering the analysis of the previously discussed XAS study. XAS measurements performed for Fe/NiO/MgO(10 Å)/Cr heterostructure revealed that the NiO spins are aligned within the surface plane under a compressive strain due to the pseudomorphic growth of thin MgO on the Cr buffer (see Fig. 4e for a schematical illustration of spin structure in Fe/NiO/Cr/MgO). For Fe/NiO/MgO(20 Å)/Cr we postulated the existence of both in-plane domains and domains with a significant out-of-plane component. In principle, the out-of-plane domains should give rise to a magnetic contrast in XMLD-PEEM image acquired with X-ray polarization along the surface normal. In our study, we did not observe a noticeable magnetic contrast in the XMLD-PEEM image acquired with the polarization axis nearly aligned to the surface normal (not shown). However, we noted a reduction of contrast in the XMLD-PEEM image recorded with in-plane X-ray polarization for Fe/NiO/MgO(200 Å)/Cr compared to Fe/NiO/MgO(10 Å)/Cr. While for NiO grown on thin MgO buffer the contrast was estimated to be about 0.66%, it was reduced to 0.55% for Fe/NiO/MgO(200 Å). The attenuation of XMLD-PEEM contrast for an image collected with in-plane X-ray polarization can be explained by the existence of depth-dependent domain structure in Fe/NiO/MgO(200 Å). In principle, PEEM measurements performed with X-ray polarization along the surface normal do not produce XMLD contrast between the nearly out-of-plane domains nor between the in-plane dominant domains. On the other hand, one can expect to observe a contrast between coexisting in-plane and out-of-plane domains. In order to understand the lack of contrast, we remind that the change of spin orientation in NiO caused by the increase of MgO thickness in Fe/NiO/MgO(*d*_*MgO*_)/Cr is induced by the competition between exchange coupling to the ferromagnet at the Fe/NiO interface and the strain–induced anisotropy at the bottom, NiO/MgO, interface. Different interactions at the top and bottom interfaces can lead to the formation of depth-dependent magnetic domain structure with in-plane domains located at the Fe/NiO interface and domains with an out-of-plane component at the NiO/MgO interface (see Fig. 4f for a schematical illustration of spin structure in Fe/NiO/MgO/Cr). The existence of similar depth-resolved antiferromagnetic domains was previously postulated for NiO film in the CoO/NiO(21 Å)/MgO system^50^. Indeed, such depth-dependent AFM domains would not give rise to XMLD contrast with out-of-plane X-ray polarization, as the surface would be homogeneously covered with in-plane AFM domains.

**Figure 5:**
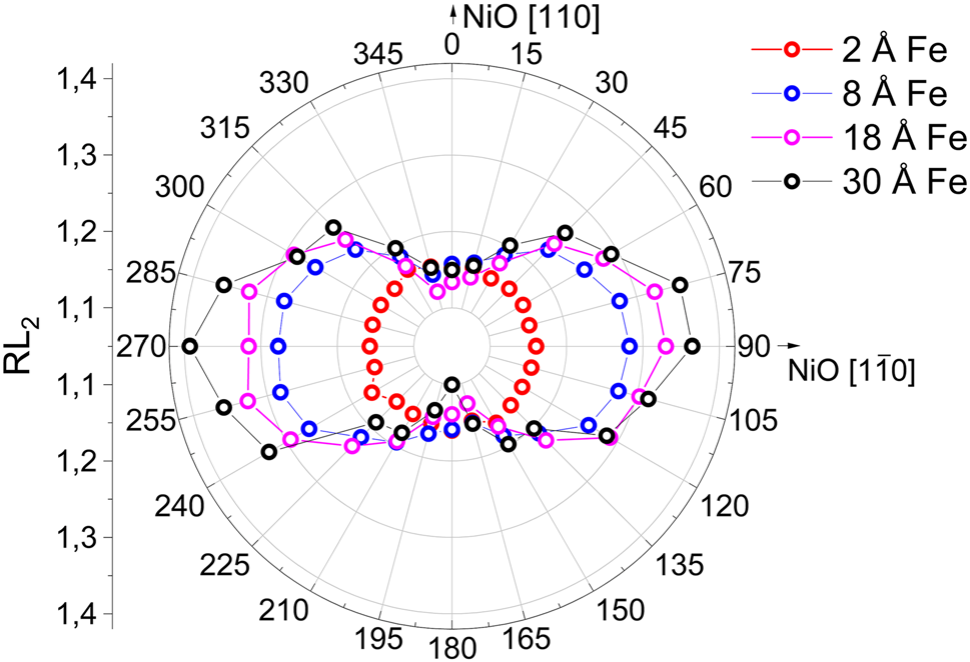
Ni RL_2_ as a function of azimuthal angle φ for Fe(*d*_*Fe*_)/NiO/MgO(85 Å)/Cr. The legend indicates the thickness of Fe.

To further investigate the influence of Fe magnetic properties on the NiO spin-axis, we performed a systematic study of NiO spin structure in Fe/NiO/MgO(85 Å)/Cr/MgO(001) for different Fe thicknesses (*d*_*Fe*_) (Fig. 5). We decided to choose *d*_*MgO*_ = 85 Å as for this MgO thickness we noted negligible ΔRL_2_ (Fig. 1c), hence negligible anisotropy of NiO between in-plane and out-of-plane orientations. Before the XMLD experiment, we magnetized the sample along the Fe[100](NiO[110]) easy axis. For 2 Å Fe layer, no change in RL_2_(φ) dependence was observed. Isotropic RL_2_ (φ) dependency can be reproduced in the simulation in which all bulk-like NiO domains are considered (Figure S4, Supplemental Material). For such a thin Fe film, the lack of XMCD at the Fe L_3_ and L_2_ absorption edges (not shown) confirmed that Fe is in a non-magnetic state. Thus, there is no FM/AFM exchange interaction that could contribute to the in-plane anisotropy in the NiO layer. For *d*_*F*e_ ≥ 8 Å, however, we noted well-defined in-plane anisotropy originating from the orthogonal coupling at the FM/AFM interface. Importantly, the in-plane anisotropy of NiO gets more pronounced with increasing Fe thickness. A gradual increase in the magnitude of RL_2_ for φ = 90° observed in the experiment can be understood if we consider growth contribution from [1-10] domain at the expense of the rest of bulk domains. The results show that the proper choice of Fe and MgO thickness allows tuning the magnetic anisotropy of the antiferromagnetic NiO.

## Conclusions

In summary, we showed how interface-induced strains and exchange interactions at an interface with a ferromagnet can be exploited to tune the magnetic anisotropy in antiferromagnetic NiO. For a NiO layer grown on a wedge-shaped MgO underlayer, the magnetic anisotropy of NiO can be modulated by exerting appropriate strain from its bottom interface. Such strain engineering was additionally combined with FM/AFM exchange coupling to create a multiple domain structure in the NiO thin film. Finally, it was shown that the repopulation of NiO domains could be induced by adjusting the thickness of the Fe layer.

## Supplementary Information

Supplementary Information 1.

## Data Availability

The data that support the findings of this study are available from the corresponding author upon reasonable request.
