# Supplementary material for: Tunable magnetic anisotropy of antiferromagnetic NiO in (Fe)/NiO/MgO/Cr/MgO(001) epitaxial multilayers
Source: Sci Rep. 2023 Mar 24;13:4824. doi: 10.1038/s41598-023-31930-z (PMC10039026; doi:10.1038/s41598-023-31930-z)
Supplement: Supplementary file 1 — Supplementary Information 1. [file 41598_2023_31930_MOESM1_ESM.pdf]

Tunable magnetic anisotropy of antiferromagnetic NiO in (Fe)/NiO/MgO/Cr/MgO(001) epitaxial multilayers

W. Janus [1)*], T. Ślęzak [1)], M. Ślęzak [1)], M. Szpytma [1)], P. Dróżdż [1)], H. Nayyef [1)], A. Mandziak [3)], D. Wilgocka – Ślęzak [2)], M. Zając [3)], M. Jugovac [4)], T. O. Menteş [4)], A. Locatelli [4)] and A. Kozioł-Rachwał [1)]

1) AGH University of Science and Technology, Faculty of Physics and Applied Computer Science, Kraków, Poland

2) Jerzy Haber Institute of Catalysis and Surface Chemistry Polish Academy of Sciences, Krakow, Poland

3) SOLARIS National Synchrotron Radiation Centre, Jagiellonian University, Krakow, Poland

4) Elettra-Sincrotrone Trieste S.C.p.A., Basovizza, Trieste, Italy

* corresponding author: wjanus@agh.edu.pl

Growth and structural properties of Fe/NiO/MgO/Cr

Epitaxial growth of Fe on NiO was confirmed by LEED measurements (Fig. S1 (a) and (b)). Figure S1 (c) and (d) show a ball model of Fe/NiO(001) interface and a sketch of the measurement configuration with relation between γ, φ angles and crystallographic directions of Fe and NiO, respectively.

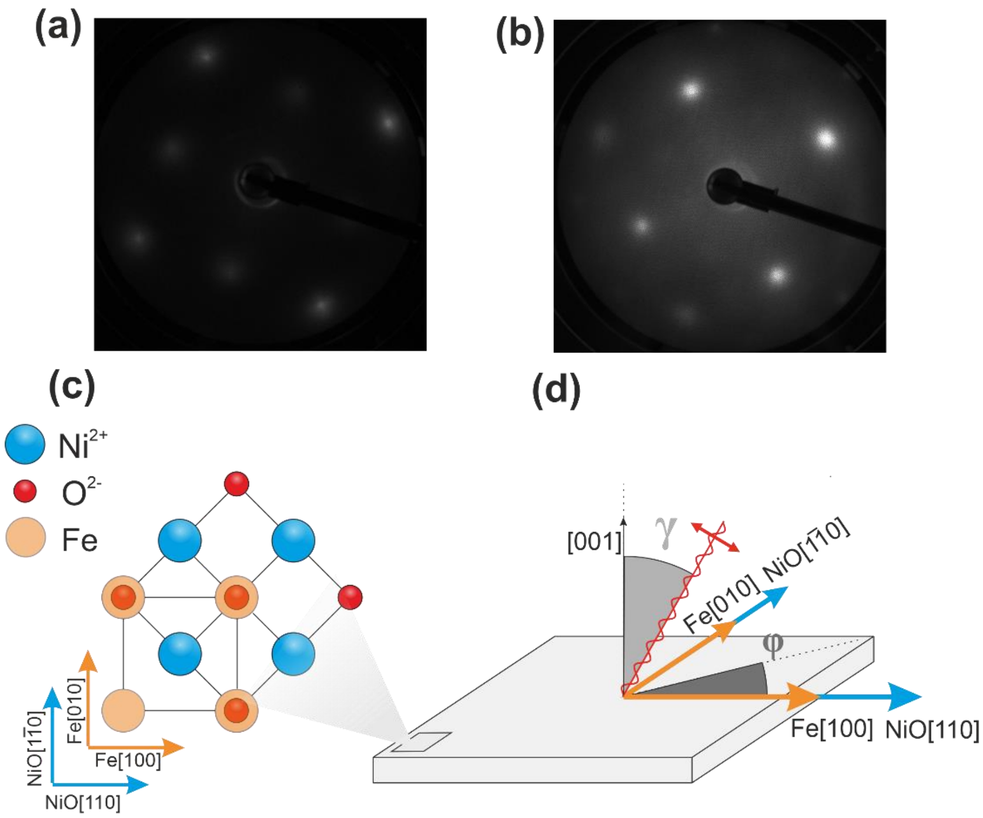

Figure S1 LEED patterns of (a) NiO(001)/Cr/MgO(001) collected at 86 eV and (b) Fe(001)/NiO(001) collected at 169 eV. (c) Ball model of Fe/NiO interface with relative Fe and NiO in-plane directions (d) Sketch of the measurement configuration with relation between γ, φ angles and crystallographic directions of Fe and NiO.

XAS measurements - Fe/NiO/Cr/MgO(001)

Figure S2 shows XAS spectra around L_2 and L_3 absorption edges of Fe measured for two opposite circular polarizations (σ+ and σ-) at φ = 0° and φ = 90° for Fe/NiO/Cr/MgO(001) and Fe/NiO/MgO/Cr/MgO(001) systems. The spectra were measured after magnetizing the sample along Fe[100] direction. For both presented systems we noted a strong polarization dependence of the spectra for φ = 0° (Fe[100]) (Fig. S3 (a) and (c), black and red curve). However, for φ = 90° (Fe[010]) we did not observe change in the shape of the spectra measured for σ+ and σ- (Fig. S2 (b) and (d), black and red curve). These results indicate that Fe magnetic moments are collinear with applied magnetic field.

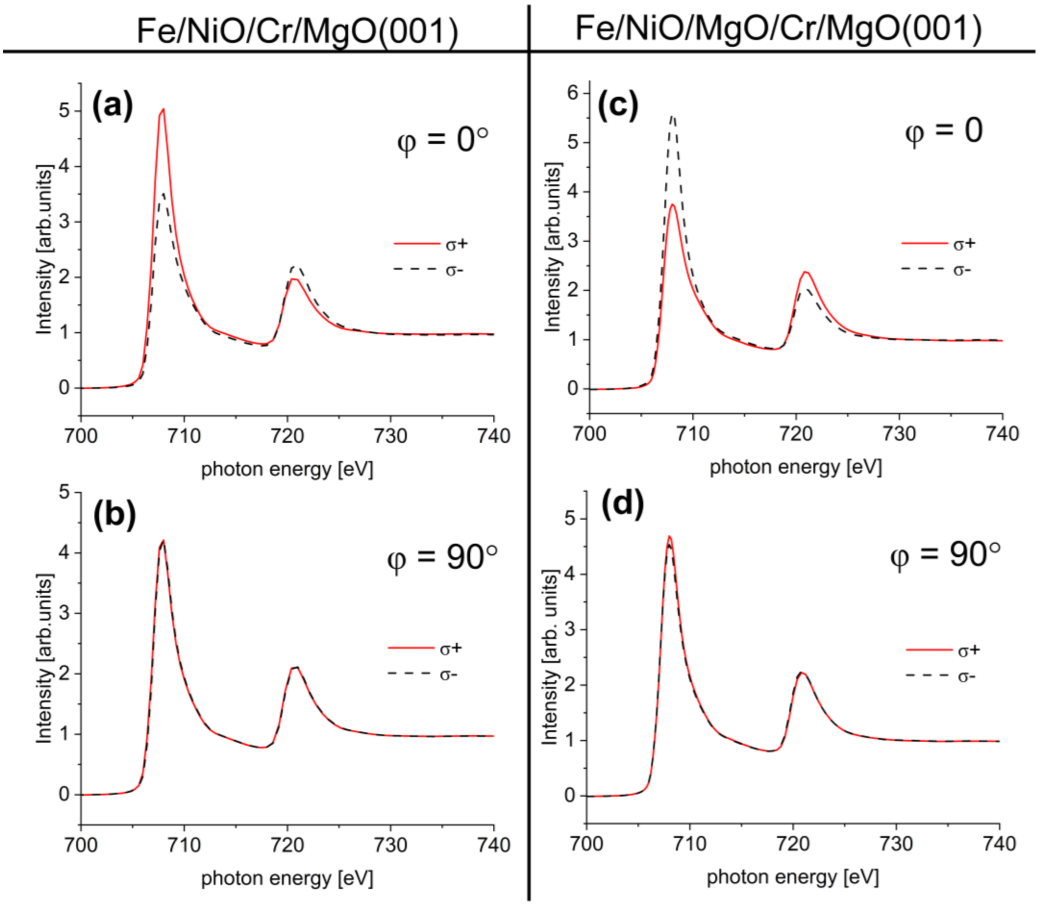

Figure S2 XAS spectra at the Fe L_2 and L_3 edges acquired for two opposite helicities of circularly polarised light (σ+, σ−) for Fe/NiO/Cr/MgO(001) (a, b) and Fe/NiO/MgO/Cr/MgO(001) (c, d), recorded at φ = 0° (a, c) and φ = 90° (b, d).

Simulation of XAS spectra

To find theoretical spectra for a given relative orientation of the polarization vector **E** and the spins axis **S**, we performed atomic multiplet calculations for NiO(001) using Crispy frontend [49]. Crispy is a graphical user interface for calculating core-level spectra using the Quanty language. Quanty is based on the model, which assumes that the X-ray absorption spectra for a given spin direction **S** and polarization **E** can be found by the relation:

$$\mu_{XAS} = -\frac{4\pi c}{\omega} Im(E^* \cdot \hat{\varepsilon} \cdot E)$$

where $E = (E_x, E_y, E_z)$ is the polarization unit vector, and $\hat{\varepsilon}$ is the dielectric tensor [52]. Since the XMLD effect in NiO is anisotropic, the approximate dielectric tensor can be written as:

$$\hat{\varepsilon}_{MLD} = \begin{bmatrix} \varepsilon_\perp + (\varepsilon_\parallel - \varepsilon_\perp)x^2 & -(\varepsilon_\parallel - \varepsilon_\perp)xy & -(\varepsilon_\parallel - \varepsilon_\perp)zx \\ -(\varepsilon_\parallel - \varepsilon_\perp)xy & \varepsilon_\perp + (\varepsilon_\parallel - \varepsilon_\perp)y^2 & -(\varepsilon_\parallel - \varepsilon_\perp)zy \\ -(\varepsilon_\parallel - \varepsilon_\perp)xz & -(\varepsilon_\parallel - \varepsilon_\perp)yz & \varepsilon_\perp + (\varepsilon_\parallel - \varepsilon_\perp)z^2 \end{bmatrix}$$

where $\varepsilon_\parallel(\varepsilon_\perp)$ is the XAS spectrum obtained for the high symmetry case S ∥ E ∥ C_4^z (C_4^x ∥ S ⊥ E ∥ C_4^z). The C_4 axis is one of the fourfold high-symmetry axes defined by the cubic point group, which correspond to the x, y, and z directions. The spectra were simulated for the atomic $2p^6 3d^8 \rightarrow 2p^5 3d^9$ transition in O_h crystal-field splitting of 10Dq = 1.4 eV [48] and a total effective exchange field felt by the Ni^{2+} ion of 6 × 27 meV [4]. Simulated NiO XAS spectra were used to calculate the L_2 ratio for selected spin orientation **S** and polarization direction **E**.

Figure S3 shows calculated polar $RL_2(\gamma)$ and azimuthal $RL_2(\varphi)$ dependencies for all possible bulk NiO domains.

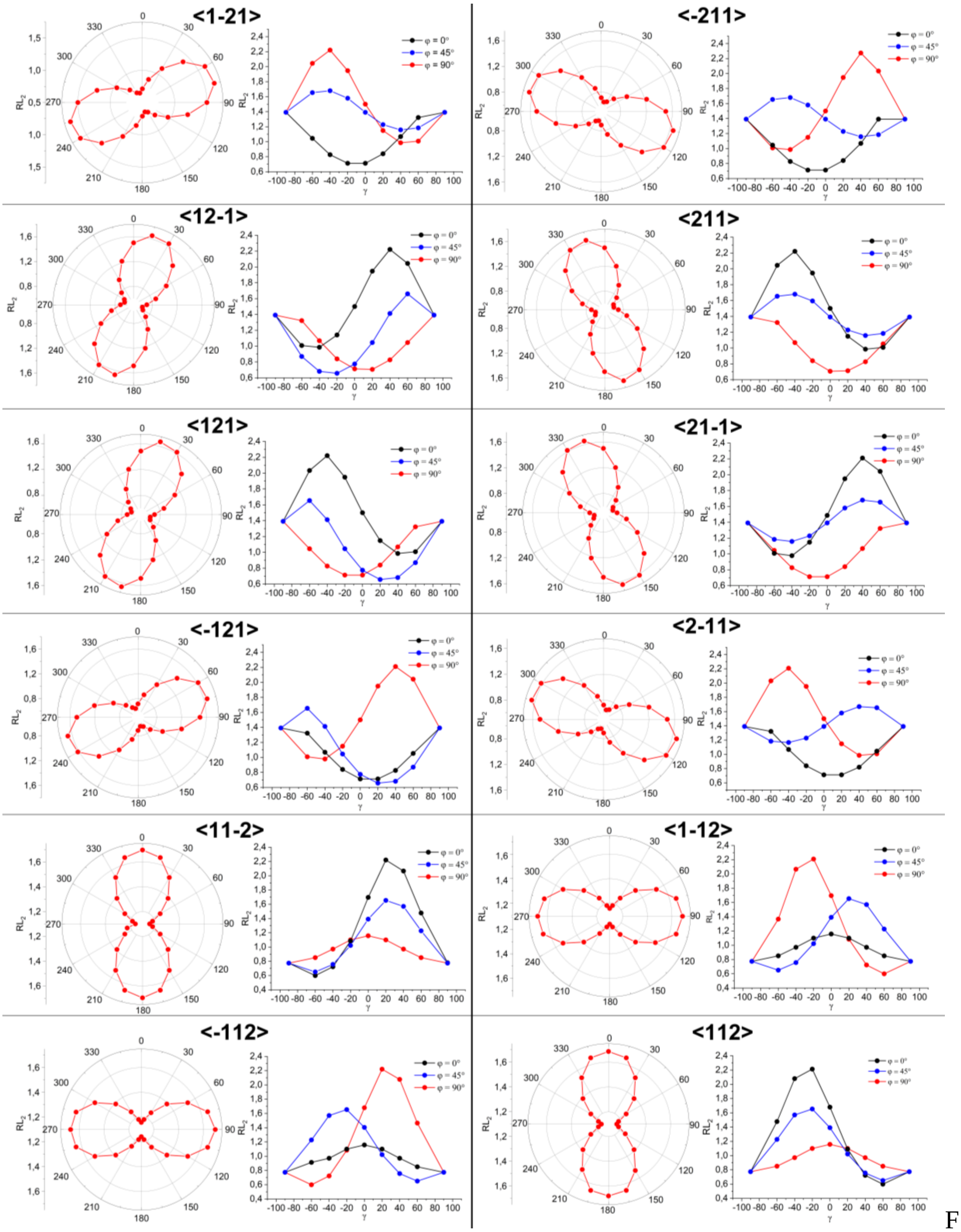

F

Figure S3 Calculated polar $RL_2(\gamma)$ and azimuthal $RL_2(\varphi)$ dependencies for all possible bulk NiO domains. The azimuthal $RL_2(\varphi)$ dependencies were calculated for $\gamma = 0°$. For all presented dependencies $\varphi = 0°$ corresponds to polarization vector **E** ‖ NiO[110] direction, and $\varphi = 90°$ corresponds to **E** ‖ NiO[1-10] direction.

NiO spin structure in Fe/NiO/MgO(85Å)/Cr/MgO(001) for different Fe thicknesses (d_{Fe})

To investigate the influence of Fe magnetic properties on the NiO spin-axis, we performed a systematic study of NiO spin structure in Fe/NiO/MgO(85Å)/Cr/MgO(001) for different Fe thicknesses (d_{Fe}). For 2 Å Fe layer, no change in $RL_2(\varphi)$ dependence was observed. Isotropic RL_2 (φ) dependency can be reproduced in simulation in which all bulk-like NiO domains are considered (Fig. S4, Supplemental Material). For $d_{Fe} \geq 8$ Å, we noted well-defined in-plane anisotropy, originating from dominant contribution from [1-10] domain.

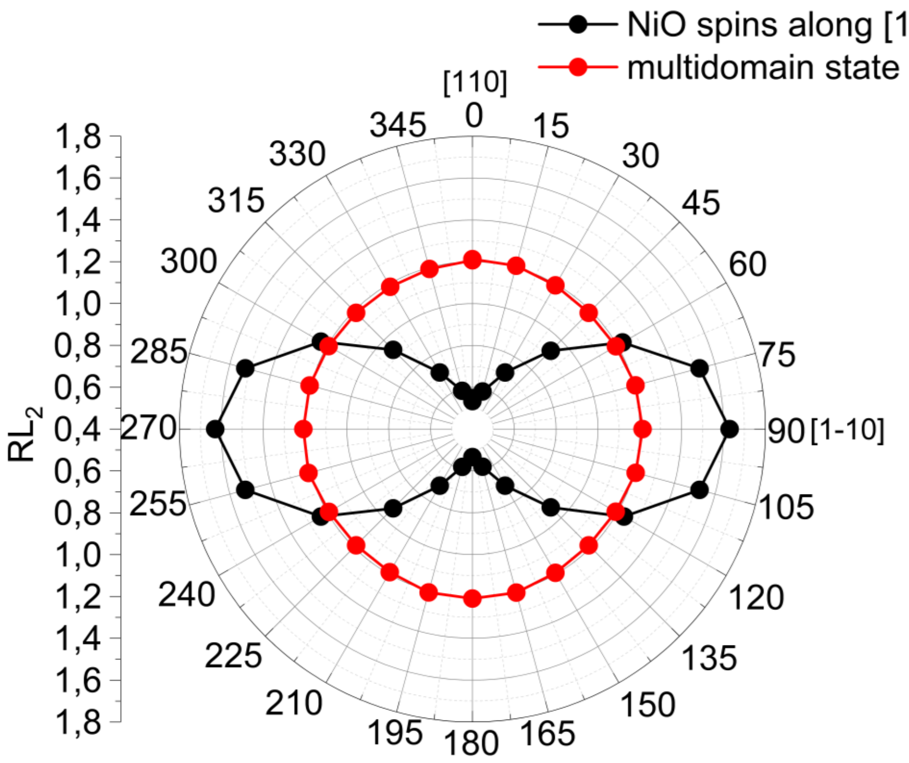

Figure S4 Calculated $RL_2(\varphi)$ dependencies obtained for all 12 bulk-like NiO domains (black line) and [1-10] in-plane NiO domain (red line).

REFERENCES

52. Krug, I. P. *et al.* Impact of interface orientation on magnetic coupling in highly ordered systems: A case study of the low-indexed Fe3 O4 /NiO interfaces. *Phys. Rev. B* **78**, 064427 (2008).
